# Clinical and pathophysiologic aspects of ECMO-associated hemorrhagic complications

**DOI:** 10.1371/journal.pone.0240117

**Published:** 2020-10-13

**Authors:** Konstantin A. Popugaev, Sergey A. Bakharev, Kirill V. Kiselev, Alexander S. Samoylov, Nikolay M. Kruglykov, Sergey A. Abudeev, Sergey V. Zhuravel, Aslan K. Shabanov, Thomas Mueller, Stephan A. Mayer, Sergey S. Petrikov

**Affiliations:** 1 Department of Intensive Care, Sklifosovsky Research Institute of Emergency Medicine of the Moscow Healthcare Department, Moscow, Russia; 2 Department of Intensive Care, State Research Center—Burnasyan Federal Medical Biophysical Center of Federal Medical Biological Agency, Moscow, Russia; 3 Department of Statistics and Cybernetics, Pirogov Russian National Research Medical University, Moscow, Russia; 4 Department of Internal Medicine II, University Hospital Regensburg, Regensburg, Germany; 5 Department of Neurology, Henry Ford Hospital, Detroit, MI, United States of America; Maastricht University Medical Center, NETHERLANDS

## Abstract

Extracorporeal membrane oxygenation (ECMO) is increasingly used to treat severe cases of acute respiratory or cardiac failure. Hemorrhagic complications represent one of the most common complications during ECMO, and can be life threatening. The purpose of this study was to elucidate pathophysiological mechanisms of ECMO-associated hemorrhagic complications and their impact on standard and viscoelastic coagulation tests. The study cohort included 27 patients treated with VV-ECMO or VA-ECMO. Hemostasis was evaluated using standard coagulation tests and viscoelastic parameters investigated with rotational thromboelastometry. Anticoagulation and hemorrhagic complications were analyzed for up to seven days depending on ECMO duration. Hemorrhagic complications developed in 16 (59%) patients. There were 102 discrete hemorrhagic episodes among 116 24-hour-intervals, of which 27% were considered to be clinically significant. The highest number of ECMO-associated hemorrhages occurred on the 2^nd^ and 3^rd^ day of treatment. Respiratory tract bleeding was the most common hemorrhagic complication, occurring in 62% of the 24-hour intervals. All 24-hours-intervals were divided into two groups: “with bleeding” and “without bleeding”. The probability of hemorrhage was significantly associated with abnormalities of four parameters: increased international normalized ratio (INR, sensitivity 71%, specificity 94%), increased prothrombin time (PT, sensitivity 90%, specificity 72%), decreased intrinsic pathway maximal clot firmness (MCFin, sensitivity 76%, specificity 89%), and increased extrinsic pathway clot formation time (CFTex, sensitivity 77%, specificity 87%). In conclusions, early ECMO-associated hemorrhagic complications are related to one traditional and two novel viscoelastic coagulation abnormalities: PT/INR elevation, reduced maximum clot firmness due to intrinsic pathway dysfunction (MCFin), and prolonged clot formation time due to extrinsic pathway dysfunction (CFTex). When managing hemostasis during ECMO, derangements in PT/INR, MCFin and CFT_ex_ should be focused on.

## Introduction

Extracorporeal membrane oxygenation (ECMO) is a technique increasingly used in the practice of intensive therapy for extracorporeal gas exchange and/or circulatory support in patients with acute respiratory and/or cardiac failure, when conventional treatment modalities are ineffective [1]. Blood flow within an artificial extracorporeal circuit will lead to thromboembolic events and necessitates therapeutic anticoagulation. Obligatory use of anticoagulants, however, will increase the risk of hemorrhagic complications, including massive life-threatening bleedings [2]. In critically ill patients, even before ECMO, many hemostatic disturbances can develop, which can be aggravated during ECMO. Therefore, prevention and correction of hemostatic disturbances during ECMO is important. The effectiveness of hemostasis management can often determine the clinical outcome in a patient in need of ECMO [3].

Traditional laboratory coagulation parameters assess separated parts of hemostasis in blood samples after centrifugation, which makes it difficult to evaluate the coagulation process as a whole [4]. Global viscoelastic methods for assessing hemostasis, including thromboelastography (TEG) and rotational thromboelastometry (ROTEM), may offer important advantages to traditional clotting tests [5]. TEG and ROTEM assess quantitative and qualitative aspects of whole blood coagulation, including clot formation, retraction, and lysis. Analysis of TEG and ROTEM data may allow intensivists to determine the mechanism of hemostatic disturbances and select a targeted therapy more accurately [6].

The purpose of this research was to better understand the pathophysiological mechanisms that underlie ECMO-associated hemorrhagic complications, and to identify traditional laboratory coagulation and ROTEM parameters that might be useful to monitor to prevent such complications during ECMO.

## Materials and methods

27 consecutive adult patients on ECMO were included into the study from June 2017 to April 2019 and all data were recorded prospectively. Inclusion criteria were age > 18 years on either veno-venous (VV) or veno-arterial (VA) ECMO, patients with proven brain death were excluded. All patients were treated at the Anesthesia, Intensive Care and ECMO Center of the State Research Center − Burnasyan Federal Medical Biophysical Center of the Federal Medical Biological Agency of Russia. The need for written patient consent was waived by the ethics committee of the hospital because laboratory investigations were conducted according to the local standard. The study was approved by the ethics committee of Burnasyan Federal Medical Biophysical Center (protocol #33, issued on 14.06.2017).

The principles of laboratory monitoring and patient management were standard, and corresponded to national and international guidelines [7–12]. ECMO was performed using RotaFlow or Cardiohelp devices (all Getinge, Rastatt, Germany). For both VV-ECMO and VA-ECMO a drainage cannula (21–25 Fr) was placed in the inferior vena cava via the femoral vein. For VV-ECMO the return cannula (17–21 Fr) was placed into the superior vena cava/right atrium via the right internal jugular vein. For VA-ECMO, the return cannula (19–23 Fr) was placed into the abdominal aorta via the femoral artery. A femoral distal perfusion cannula was placed in all VA-ECMO cases.

All patients received unfractionated heparin for anticoagulation. Immediately after cannulation all patients received a bolus of heparin of 50–100 U/kg, thereafter its dose was titrated using the activated clotting time (ACT), the activated partial thromboplastin time (aPTT), and the clotting time in the intrinsic thromboelastometry (INTEM) mode (CTin). Target values for ACT were 140–160 sec, for aPTT 45–55 sec, and for CTin 240–260 sec. APTT and ACT were monitored at least every 8 hours, CTin every 24 hours. Antithrombin III (AT-III), fibrinogen, and ROTEM with TEG Rotem Delta (TEM International GmbH, Munich, Germany) were examined on a daily basis, according to our standard management protocol. The international normalized ratio (INR), prothrombin time (PT), prothrombin index (PTI) were monitored daily, as well as clinical and biochemical blood tests, procalcitonin test (PCT), and C-reactive protein (CRP). A list of all monitored traditional and viscoelastic coagulation parameters together with target therapeutic values (Table 1) and interventions in case of deviation (Table 2) are provided in the online supplement. Hemorrhagic complications were assessed daily and classified as follows: (1) localization–nosebleed, gastrointestinal bleeding (GI bleeding), hematuria, bleeding from cannula sites, pulmonary hemorrhage, hematothorax, intracranial hemorrhage, retroperitoneal hemorrhage; (2) severity of bleeding–a) the absence of hemorrhagic complications; b) clinically insignificant bleeding (to stop bleeding, soft tissue infiltration is sufficient with a local anesthetic solution with vaso-constricting drugs, local tissue cooling, or anterior nasal tamponade); c) clinically significant bleeding (resulting in a decrease in hemoglobin of ≤ 2 mg/dl within 24 hours, but requiring surgical or endoscopic hemostasis, or posterior nasal tamponade); and d) major bleeding (a decrease in hemoglobin of > 2 mg/dl making it necessary to perform surgical or endoscopic hemostasis and hemorrhage of any volume in the retroperitoneal or intracranial space) [13].

**Table 1:** Overview of monitored traditional and viscoelastic coagulation parameters with target therapeutic values.

Parameter	Target value	Parameter	Target value
Hb, g/l	> 100	ACT, sec	140–160
Platelets, 10^9^/L	> 80.000	CTex, sec	38–79
aPTT, sec	45–55	CFTex, sec	34–159
INR	0.85–1.15	MCFex, mm	50–72
PT, sec	13–18	CTin, sec	240–260
Fibrinogen, g/l	1.8–4	CFTin, sec	30–110
AT-III, %	80–120	MCFin, mm	50–72
PTI, %	70–130	MCFex-MCFfib, mm	> 30
		MCEex-MCEfib, mm	> 142

Abbrev.: aPTT—activated partial thromboplastin time; AT–III–antithrombin III; PTI—prothrombin index; INR- international normalized ratio; PT—prothrombin time; ACT—activation coagulation time; CTin—coagulation time intem; CFTin—clot formation time intem; MCFin—maximum clot firmness intem; CTex—coagulation time extem; CFTex—clot formation time extem; MCFex—maximum clot firmness extem; MCF_fib—_maximum clot firmness fibtem; MCEfib—maximum clot elasticity.

**Table 2:** Therapeutic interventions to correct pathological coagulation parameters.

Laboratory scenario	Correction
Platelets <80.000–100.000/μl, decreased MCFex, MCFex-MCFfib <30, MCEex-MCEfib <142	Platelet concentrate
Decreased PTI and/or an increase in CTex> 79 sec.	fresh frozen plasma, prothrombin complex concentrate
Decreased fibrinogen, decreased MCFfib	Cryoprecipitate, fibrinogen concentrate
Difference between ARTEM/EXTEM-A10 more than 10%	Tranexamic acid
Decreased Antithrombin III less than 60%	AT-III

Note: MCFex—maximum clot firmness extem; MCF_fib—_maximum clot firmness fibtem; MCEfib—maximum clot elasticity.

Statistical analysis was performed using IBM SPSS Statistics v 23 software. Significance of differences between groups was examined using the Chi-square test. The normality test of quantitative indicators was carried out using the Shapiro-Wilk test. The sensitivity and specificity of the methods was determined using ROC-curves.

For statistical analysis, the period of ECMO in all observations was divided into up to seven 24-hour-intervals per patient. Each 24-hour-interval began with the measurement of all viscoelastic and traditional coagulation parameters.

## Results and discussion

In accordance with the inclusion criteria 27 patients were included in the study: 19 VV-ECMO cases and 8 VA-ECMO cases. The cause of respiratory failure requiring VV-ECMO was community-acquired pneumonia in 18 (67%) cases and neurogenic pulmonary edema in one patient after aneurysmal subarachnoid hemorrhage. Acute myocardial infarction was the cause of cardiac failure in all 8 (30%) VA-ECMO cases. The duration of ECMO was 1–33 (mean 8.0 ± 7.2) days and length of stay (LOS) in the ICU was 1–47 (18 ± 8.4) days (Table 3). The duration of ECMO was less than 7 days in eight patients. Mortality was 56% (N = 15, N = 8 for VV-ECMO (42.1%), N = 6 (75%) for VA-ECMO). A summary of demographic data and complications is provided in Tables 3 and 4. Acute renal failure (N = 18, 67%), hemorrhagic complications (N = 16, 59%) and sepsis (N = 11, 41%) were the most common complications.

**Table 3:** Demographic data.

Number of patients	27
Male/Female	18/9
Age, years	47 ± 15, 23–84
Weight, kg	79 ± 19, 54–120
Duration of mechanical ventilation before VV-ECMO, days	2 ± 4, 1–15
Total duration of mechanical ventilation for VV-ECMO, days	20 ± 11, 3–47
Duration of ECMO, days	8 ± 7, 1–33
LOS in the ICU, days	18 ± 8, 1–47
LOS in the hospital	27 ± 18, 1–67
Mortality	15 (56)

Data are mean ± SD, range, or N (%)

**Table 4:** Complications during hospitalization.

Renal failure	18 (67)
Hemorrhagic complications	16 (59)
Sepsis	11 (41)
Hospital-acquired pneumonia	9 (33)
Urinary tract infection	6 (22)
Liver failure	6 (22)
Hydrothorax	4 (15)
Deep vein thrombosis of the lower extremities	3 (11)

Data are N (%) of affected patients

Overall, 155 24-hour-intervals occurring within the first 7 days of ECMO were analyzed, and any bleeding was documented in 102 of these periods. Respiratory tract bleeding was the most common bleeding complication, occurring in 63 (62%) of the periods analyzed, followed by gastrointestinal (12%), ECMO cannula (6%) and tracheostomy site (6%) hemorrhage (Table 5).

**Table 5:** Bleeding complications during ECMO.

Respiratory tract bleeding		63 (62)
Gastrointestinal hemorrhage		12 (12)
ECMO Cannula site hemorrhage		6 (6)
Tracheostomy site hemorrhage		6 (6)
Hemothorax		3 (3)
Intracranial hemorrhage		1 (1)
Nose bleeding		1 (1)
Oral cavity hemorrhage		1 (1)
Combined hemorrhages	Pulmonary + Gastrointestinal hemorrhage	4 (4)
	Pulmonary hemorrhage + Hemothorax	3 (3)
	Tracheostomy cannula site + Cannula site hemorrhage	2 (2)

Data are N (%) of 1-hour monitoring periods

**Fig 1:**
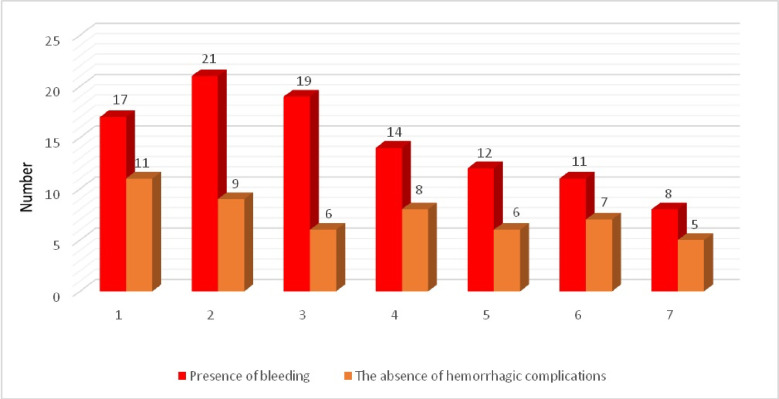
Incidence of ECMO-associated bleeding during the first 7 days of the procedure.

ECMO–associated hemorrhages occurred most often during the first 5 days of the ECMO run with a peak on the 2^nd^ and 3^rd^ day of the procedure (Fig 1).

**Fig 2:**
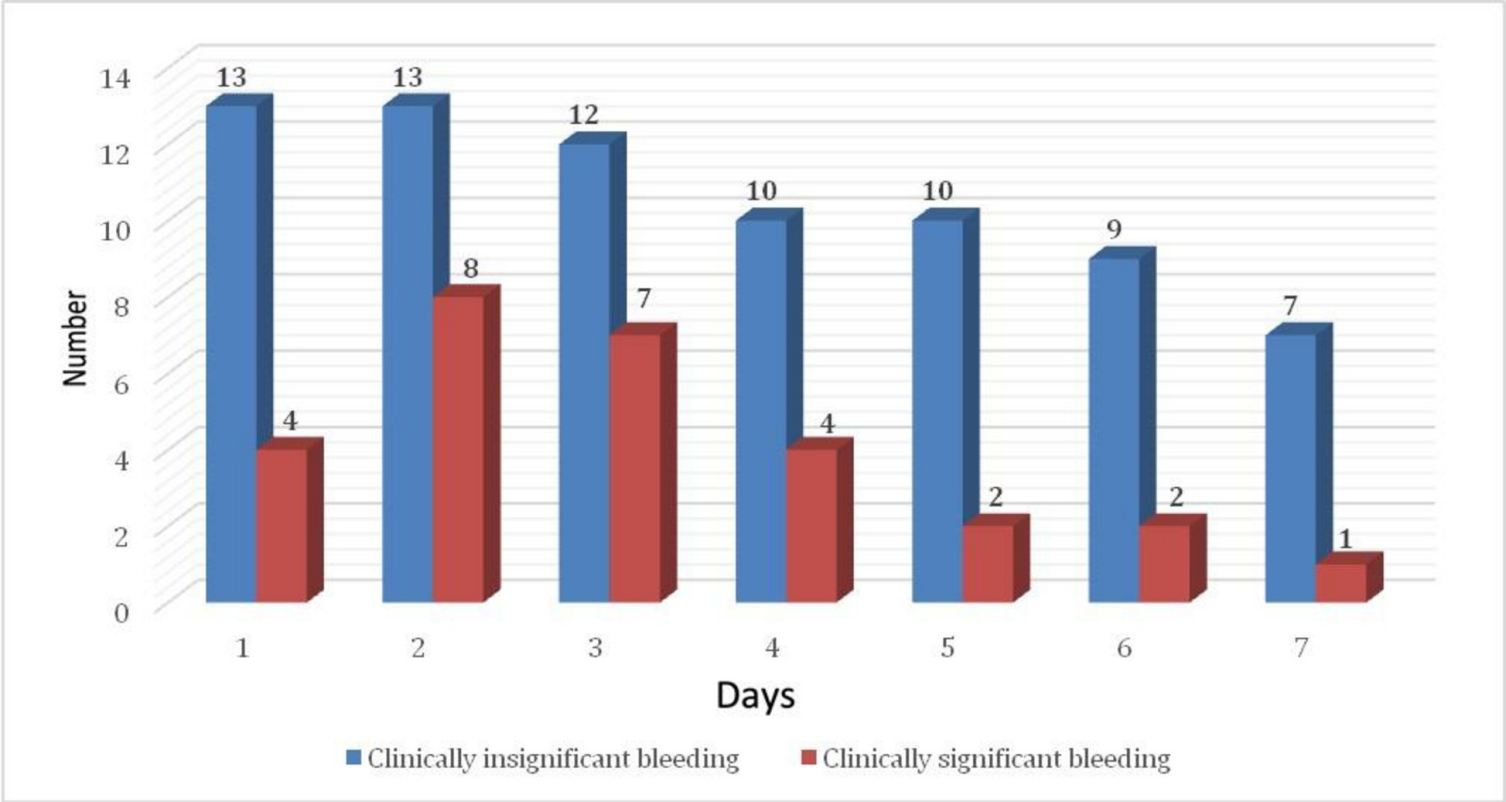
Severity of ECMO-associated bleeding during the first 7 days of the procedure.

Clinically insignificant hemorrhages took place more often than clinically significant ones: 74 (73%) and 28 (27%) events, respectively (Fig 2).

All laboratory time points were divided into two groups according to the presence or absence of bleeding during the following 24-hour-interval. Table 6 presents the 5 out of 6 traditional coagulation tests and 10 out of 15 ROTEM parameters that significantly differed between “bleeding” and “no bleeding” 24-hour intervals. Paradoxically, levels of fibrinogen were higher immediately prior to ECMO-associated bleeding episodes. While the extrinsic pathway maximal clot firmness (MCFex) during periods of ECMO-associated bleeding was significantly lower in comparison to periods without ECMO-associated bleedings, mean values of MCFex remained within the normal reference range in both groups. Sensitivity and specificity for predicting ECMO-associated bleeding were calculated for all monitored laboratory parameters which were significantly different between “bleeding” and “no bleeding” intervals (Table 6).

**Table 6:** Hemostatic parameters during 24-hours periods with or without ECMO-associated bleeding.

Laboratory parameter	Reference range	With Bleeding		Without Bleeding		*P*
		Median	IQR	Median	IQR	
aPTT sec	24–34	36.5	34.5;52.9	44.3	33.9;59.7	0,99
AT–III, %	80–120	52.1	12.6–82.5	81.1	69.5–91.6	**0.041**
INR	0.85–1.15	1.67	1.3–1.84	1.26	1.2–1.38	**0.001**
PT, sec	13–18	19.5	14.7–26.5	14.4	13.7–15.5	**0.001**
Fibrinogen, g/l	1.8–4.0	3.8	2.4–5.0	3.5	2.9–4.3	**0.01**
Platelets, /μL (K)	200–400	67	60–118	133	106–194	**0.002**
CTapt, sec	38–79	84	72.5–123.7	91	74.5–134	0.881
CFTapt, sec	34–159	143	72.5–233.2	96	68.3–134	**0.015**
MCFapt, mm	50–72	56	42.7–66.5	64	59–71.2	**0.012**
CTin, sec	120–240	233	163–334	191	161–221	**0.01**
CFTin, sec	30–110	96	74–172	88	64–140	**0.01**
MCFin, mm	50–72	63	54–66	61	54–69	**0.01**
CTex, sec	38–79	88	76–121	80	75,5–86	0.19
CFTex, sec	34–159	112	107–142	83	67–101	**0.001**
MCFex, mm	50–72	60	59–62	65	60–70	**0.017**
CFTfib	No data	133	122–333	136	35–139	0.515
MCFfib	9–25	15	16–29	25	20–29,5	**0.007**
APTEM-A10, mm	44–66	46	33–47	55	44–63	**0.032**
EXTEM-A10, mm	43–65	51	46–56	60	54–64	**0.04**
MCFex—MCFfib	< 30	40	34–43	40	30,5–45	0.96
MCEex—MCEfib	>142	127	12–137	164	117–201	**0.08**

Note: AT–III–antithrombin III; INR- international normalized ratio; PT—prothrombin time; CTapt—coagulation time aptem; CFTapt—clot formation time aptem; MCFapt—maximum clot firmness aptem; CTin—coagulation time intem; CFTin—clot formation time intem; MCFin—maximum clot firmness intem; CTex—coagulation time extem; CFTex—clot formation time extem; MCFex—maximum clot firmness extem; MCF_fib—_maximum clot firmness fibtem; MCEfib—maximum clot elasticity. *P*–means difference between “with bleeding” and “without bleeding” intervals

**Fig 3:**
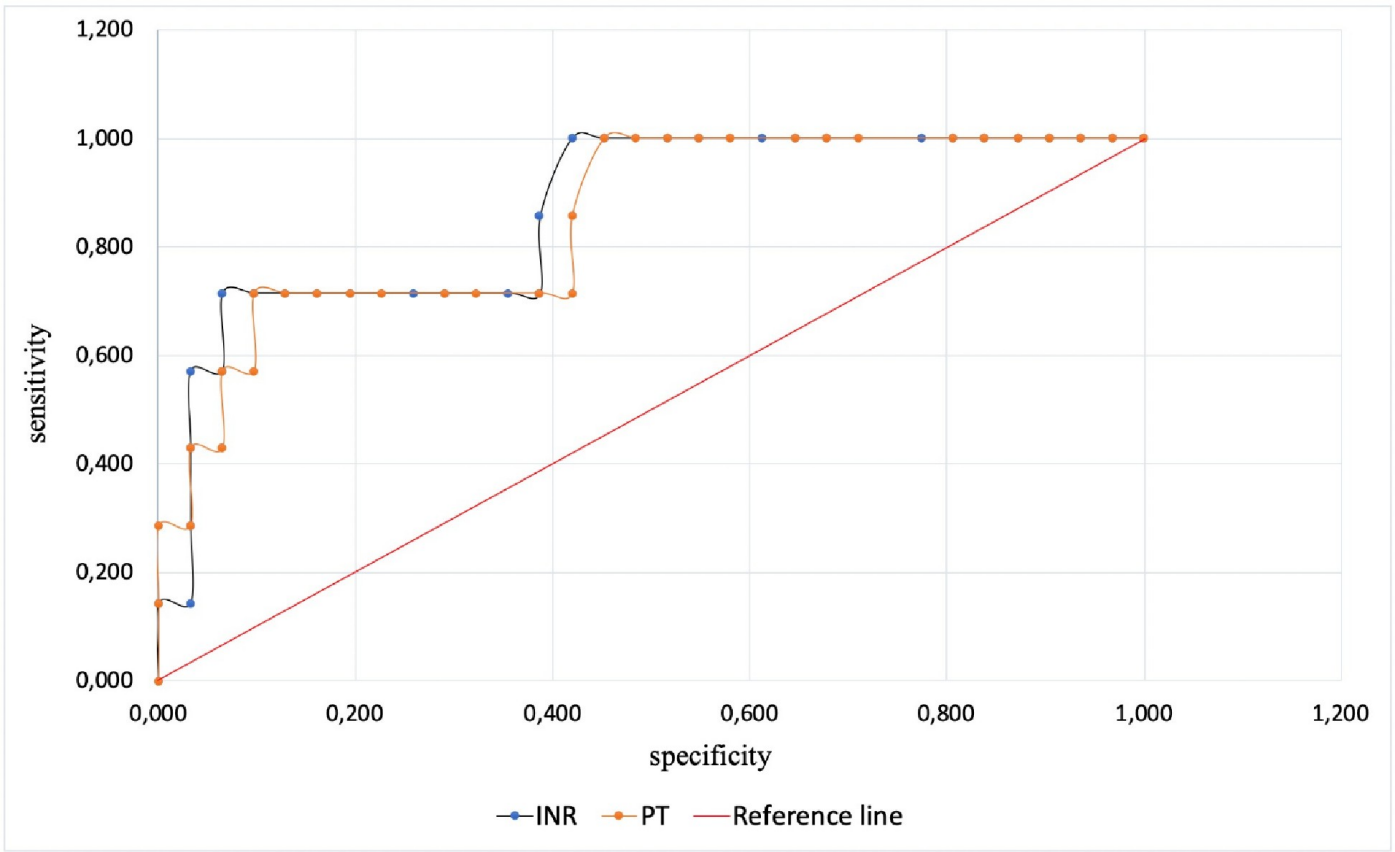
Sensitivity and specificity of INR and PT.

Among all traditional clotting tests INR (AUC 0.763, cut-off value 1.41) and PT (AUC 0.762 cut-off value 15.85) predicted ECMO-associated hemorrhagic complications best with a sensitivity of 71% and 72%, and a specificity of 94% and 90%, respectively (Fig 3). The other parameters could not accurately predict bleeding events.

**Fig 4:**
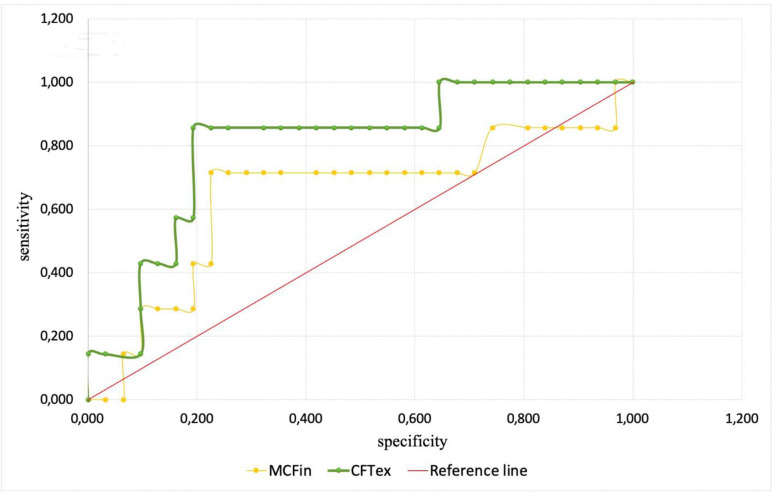
Sensitivity and specificity of MCFin and CFTex.

Among all monitored and analyzed ROTEM parameters only intrinsic pathway maximal clot firmness (MCFin, AUC 0.607, cut-off value 53.0) and extrinsic pathway clot formation time (CFTex, AUC 0.571, cut-off value 58.0) could predict ECMO-associated hemorrhagic complications for the following 24 hours with a sensitivity of 76% and 89%, and a specificity of 77% and 87%, respectively (Fig 4).

**Fig 5:**
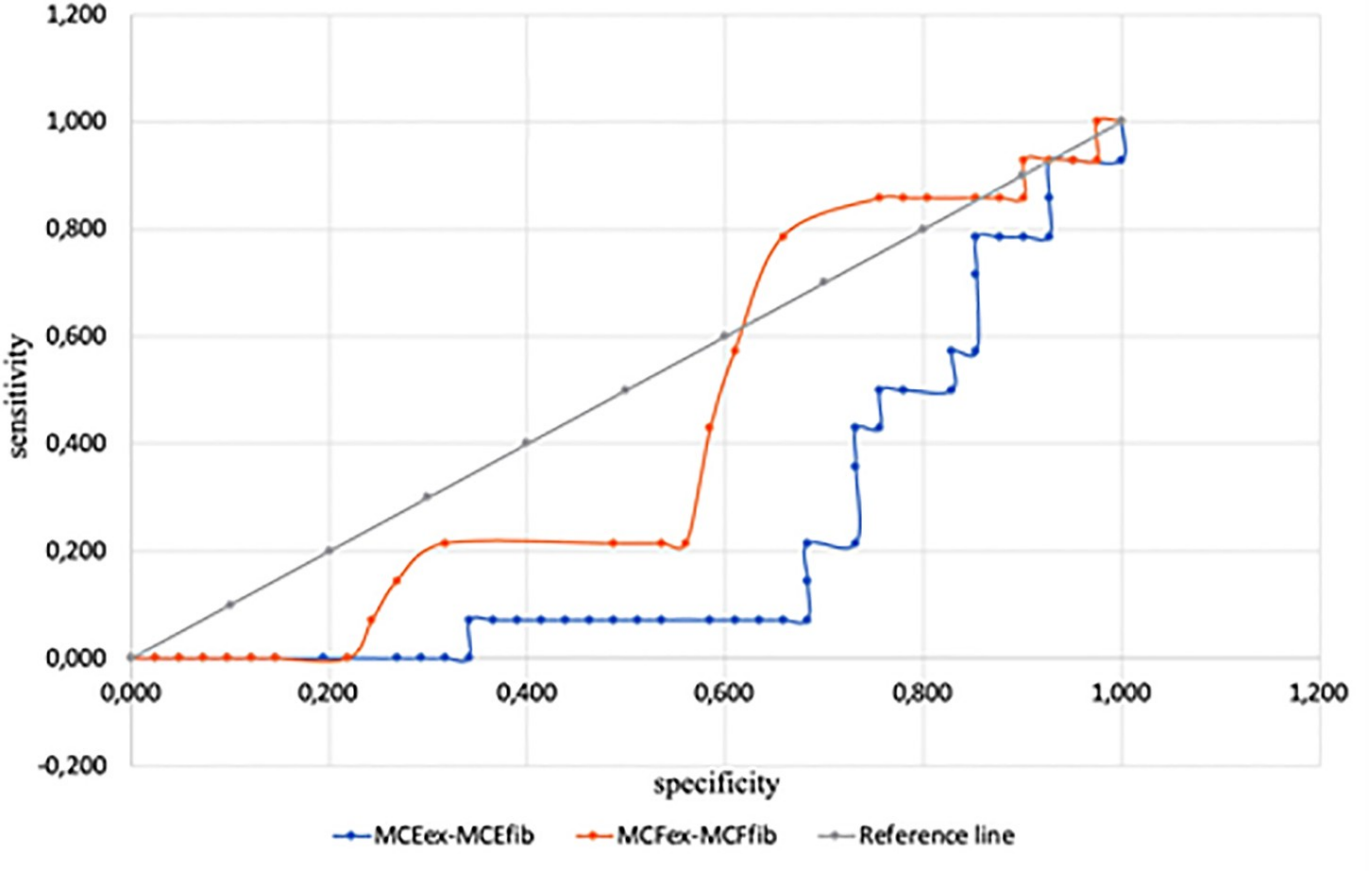
Sensitivity and specificity of MCFex-MCFfib and MCEex-MCEfib.

Sensitivity and specificity in predicting ECMO-associated hemorrhagic complications for differences of MCFex-MCFfib and MCEex-MCEfib were poorer and are provided in the online supplement. Sensitivity and specificity of MCFex-MCFfib (AUC 0.554, cut-off value 33.5) were 34% and 46%, of MCEex-MCEfib (AUC 0.532, cut-off value 79.0) 35% and 56%, respectively (Fig 5).

Hemorrhagic and thromboembolic complications in patients on ECMO are common and contribute to organ dysfunction and unfavorable outcomes [4]. Our results show that ECMO-associated bleeding can develop in more than 50% of patients and are associated with a wide range of coagulation derangements [14–16].

It has to be taken into account, that patients on VV-ECMO or on VA-ECMO are fundamentally different from a clinical point of view. For example, septic patients with community acquired pneumonia on VV-ECMO may present with disseminated intravascular coagulopathy, whereas patients with cardiogenic shock after myocardial infarction may have received platelet inhibitory therapy. Also, due to different pathophysiological mechanisms the incidence in cerebral bleeding is unlike in VA- and VV-ECMO, but played only a minor part in our cohort (one patient). Yet, apart from different cannulation strategies and somewhat increased pump-speed for increased afterload in VA-ECMO, the principal components and settings of the devices are identical, and their influence on coagulation is largely comparable. During ECMO in critical care settings numerous artificial procoagulant and anticoagulant factors simultaneously influence the coagulation system. The most significant procoagulant factors during ECMO are a long-lasting blood contact with the synthetic non-endothelial surface of the ECMO circuit, endothelial damage at the site of vessel cannulation, endothelial dysfunction due to biologically active substances, decreased blood flow between the vessel wall and the cannula, the procoagulant effects of free hemoglobin, an ECMO flow of less than 2 L/min, disseminated intravascular coagulation (DIC), heparin-induced thrombocytopenia, thrombin hypersecretion due to activation of white blood cells and the complement system, heparin resistance, and many more [17–20]. The most significant anticoagulant factors during ECMO are anticoagulation with heparin, thrombocytopenia or thrombocytopathy, decreased concentrations of coagulation factors, including fibrinogen and factor XIII, shear stress phenomenona, acquired Von Willebrand syndrome among others [21, 22].

At any given time during an ECMO run, a unique combination of pro- and anticoagulant factors are activated. Whether thrombotic or hemorrhagic complications will develop depends largely on the relative balance of these factors. Our study showed that the majority of ECMO-associated bleedings developed on the 2^nd^-3^rd^ day of the procedure.

The main result of this study is the finding that two traditional coagulation and two novel viscoelastic parameter abnormalities are associated with bleeding during ECMO: PT and INR elevation, reduced maximum clot firmness related to intrinsic pathway dysfunction (MCFin), and prolonged clot formation time due to extrinsic pathway dysfunction (CFTex). To the best of our knowledge, this study is the first to use a combination of traditional laboratory coagulation parameters and ROTEM for assessment of hemostasis during ECMO.

Pathophysiologically, the PT and INR reflect the functional activity of the extrinsic pathway of coagulation, which relies mainly on four coagulation factors: FVII, FX, FV and FII [1]. All our patients received heparin, and theoretically, PT and INR could be increased due to anticoagulation with heparin [23]. However, the levels of aPTT, ACT, and the CTin level were either normal or lower than they should be if pharmacological anticoagulation with heparin is adequate. These data suggest that heparin alone cannot explain the association between increased PT/INR and ECMO-associated bleeding.

At the same time, decreased levels of FVII, FX, FV and FII during ECMO can occur due to a number of reasons, including adsorption of these proteins to the ECMO circuit, shear stress phenomena, and decreased synthesis of these factors by the liver for a variety of reasons [23, 24]. The association of increased PT/INR with ECMO-associated bleeding provides a rationale for administering fresh frozen plasma (FFP) or prothrombin complex concentrate (PCC) in ECMO patients with acute bleeding. We could not confirm the observed association between elevated aPTT and hemorrhagic complications during ECMO cited by a prior study [25].

Fibrinogen levels, determined by the classical Clauss assay, and functional fibrinogen, measured by EXTEM and FIBTEM, were within the normal range. Moreover, fibrinogen was actually higher during 1-hour periods when bleeding was present as opposed to absent, and fibrinogen paradoxically exceeded normal values in some patients with ECMO-associated hemorrhagic complications. In our cohort of patients, we did not observe that hypofibrinogenemia was a leading cause of ECMO-associated bleeding, as was previously reported [26]. At the same time fibrinogen, being an acute-phase protein, increases in almost all critical illnesses [2], and increases in fibrinogen in critically ill patients have been associated with a simultaneous decrease in FXIII levels [27]. This phenomenon may contribute to the risk of bleeding in critically ill patients in general, including those on ECMO. Furthermore, we analyzed only the first week on ECMO for bleeding episodes, and hyperfibrinolysis induced by oxygenator clotting more commonly occurs at a later stage.

Platelets play a crucial role in the adequate functioning of hemostasis, and thrombocytopenia develops frequently during ECMO. Traditional targeted platelet levels during ECMO are 80–100 thousand per μl [28]. At the same time, it is known that platelet counts above 10–20 thousand per μl are required to prevent spontaneous bleedings [29]. Platelet counts were significantly lower in our study when bleeding had occurred despite our efforts to achieve target platelet levels above 80 thousand per μl. Platelet function as well as absolute counts play a considerable role in hemostasis [30]. Thrombocytopathy can also be an immediate cause of or contribute to ECMO-related hemorrhage [28]. In our study, we assessed platelet function with a variety of measures of maximum clot firmness (MCF) and elasticity (MCE). According to the literature, platelet function most reliably is reflected by the differences in MCE_ex_-MCE_fib_ and, to a lesser extent, MCF_ex_-MCF_fib_ [31–33]. According to our data, MCF_ex_-MCF_fib_ and MCE_ex_-MCE_fib_ had only borderline sensitivity and specificity for predicting bleeding (Fig 3). To assess the function of platelet hemostasis during ECMO the best parameter to monitor most likely is MCF_ex_-MCF_fib_, with platelet transfusion an option when values fall below 142.

Another interesting result of our study is the association of reduced intrinsic pathway maximal clot firmness (MCFin) and increased extrinsic pathway clot formation time (CFT_ex_) with ECMO-associated bleeding (Fig 2). These parameters reflect the functional activity of platelets, fibrinogen, fibrin stabilizing factor XIII and von Willebrand factor [27, 31, 33]. Taking into account normal or increased levels of fibrinogen, it can be concluded that diminished platelet hemostasis and/or FXIII dysfunction and/or acquired von Willebrand syndrome is a likely mechanism of ECMO-associated hemorrhagic complications. It is possible to rule out platelet dysfunction using the MCE_ex_-MCE_fib_ parameters as described above. Routine investigation of FXIII levels and von Willebrand factor is an expensive and rather difficult task and is generally not indicated since both factors can be replaced using cryoprecipitate. Thus, when bleeding during ECMO is associated with changes in MCF_in_ and CFT_ex_, cryoprecipitates should be used.

Our study has a number of limitations that may have affected the accuracy or validity of our results. First, our study was performed in a single center, but can be used as a design template for future multicenter studies. Second, a diagnosis of sepsis was common in our patient cohort, so it is impossible to completely exclude sepsis-associated disturbances of hemostasis as a contributing factor to the hemostatic derangements that we observed. Third, we only studied our patients for the first 7 days on ECMO, so we cannot draw any conclusions about hemostatic abnormalities during longer runs. Fourth, the relatively small number of patients somewhat limits the reliability of the data obtained. The hour-to-hour design of correlating bleeding to coagulation may have led to significant over-representation of some patients over others, and by necessity patients with the most severe disease who die early are under-represented. Fifth, the accuracy of the results may have been influenced by the absence of some coagulation parameters during some of the 24-hour-intervals. Finally, conducting a separate analysis of serious bleeding complications with exclusion of minor bleedings, like respiratory tract bleeding from the analysis, would allow calculating a so-called dose-related effect of bleeding to patients’ condition and outcomes; however, such an analysis requires more patients.

It should be remembered, that coagulation alterations during ECMO are complex, and many pathophysiological events are not fully understood. There are significant differences between individual patients, both related to the underlying disease, but also to individual reactions to the ECMO circuit. Coagulation alterations will change during the course of prolonged ECMO runs, and different mechanisms may result in clotting and/or bleeding. Our study had intended to analyze the influence on and alterations of standard coagulation diagnostics used in the ICU. The importance of individual factors like FVIII, FXIII, large monomers of vWF among others remains to be elucidated yet. Along with this, our study opens avenues for future perspective research.

## Conclusions

Although a very broad array of hemostatic derangements can occur during ECMO, our preliminary data suggest that the most important pathophysiological mechanisms of ECMO-associated bleeding are related to dysfunction of factors of the extrinsic pathway of coagulation and platelet hemostasis. Specifically, our data suggest that when managing hemostasis during ECMO, a focus should be set on PT/INR, MCF_in_ and CFT_ex_. It remains to be determined if proactive administration of FFP, PCC, platelet concentrates, cryoprecipitate, or FXIII directed towards correcting various coagulation or ROTEM abnormalities can prevent the development of hemorrhagic complications.

## Supporting information

S1 Data (XLSX)
